# Lack of detection of *Candida nivariensis* and *Candida bracarensis* among 440 clinical *Candida glabrata* sensu lato isolates in Kuwait

**DOI:** 10.1371/journal.pone.0223920

**Published:** 2019-10-16

**Authors:** Mohammad Asadzadeh, Ahlam F. Alanazi, Suhail Ahmad, Noura Al-Sweih, Ziauddin Khan

**Affiliations:** Department of Microbiology, Faculty of Medicine, Kuwait University, Jabriya, Kuwait; Lebanese American University, LEBANON

## Abstract

Occurrence of *Candida nivariensis* and *Candida bracarensis*, two species phenotypically similar to *Candida glabrata* sensu stricto, in human clinical samples from different geographical settings remains unknown. This study developed a low-cost multiplex PCR (mPCR) and three species-specific singleplex PCR assays. Reference strains of common *Candida* species were used during development and the performance of mPCR and singleplex PCR assays was evaluated with 440 clinical *C*. *glabrata* sensu lato isolates. The internal transcribed spacer (ITS) region of rDNA was also sequenced from 85 selected isolates and rDNA sequence variations were used for determining genetic relatedness among the isolates by using MEGA X software. Species-specific amplicons for *C*. *glabrata* (~360 bp), *C*. *nivariensis* (~250 bp) and *C*. *bracarensis* (~180 bp) were obtained in mPCR while no amplicon was obtained from other *Candida* species. The three singleplex PCR assays also yielded expected results with reference strains of *Candida* species. The mPCR amplified ~360 bp amplicon from all 440 *C*. *glabrata* sensu lato isolates thus identifying all clinical isolates in Kuwait as *C*. *glabrata* sensu stricto. The results of mPCR were confirmed for all 440 isolates as they yielded an amplicon only in *C*. *glabrata* sensu stricto-specific singleplex PCR assay. The rDNA sequence data identified 28 ITS haplotypes among 85 isolates with 18 isolates belonging to unique haplotypes and 67 isolates belonging to 10 cluster haplotypes. In conclusion, we have developed a simple, low-cost mPCR assay for rapid differentiation of *C*. *glabrata* sensu stricto from *C*. *nivariensis* and *C*. *bracarensis*. Our data obtained from a large collection of clinical *C*. *glabrata* sensu lato isolates show that *C*. *nivariensis* and *C*. *bracarensis* are rare pathogens in Kuwait. Considerable genetic diversity among *C*. *glabrata* sensu stricto isolates was also indicated by rDNA sequence analyses.

## Introduction

*Candida* spp. are identified as the fourth most frequent cause of bloodstream infections in hospitalized patients and third common cause of central-line associated bloodstream infections in patients admitted to intensive care units (ICUs) [1, 2]. Invasive candidiasis is associated with mortality rates of ~50% [1, 2]. Nearly 90% of *Candida* infections are caused by only four species/species complexes comprising *Candida albicans*, *Candida glabrata*, *Candida parapsilosis* and *Candida tropicalis* [3]. Although *C*. *albicans* is the most commonly isolated species, >50% of all *Candida* infections are now caused by non-*albicans* species of *Candida* [2, 4–6]. *Candida glabrata* sensu stricto has emerged as the second or third most frequently isolated *Candida* species from patients with bloodstream, vulvovaginal and oral infections [2, 6–8].

The emergence of *C*. *glabrata* as a major cause of invasive fungal infections is worrisome due to high mortality rates associated with these infections, particularly in immunocompromised elderly patients requiring major surgery and neutropenic patients [2, 9]. The higher mortality rates have been attributed to the intrinsic and/or rapidly acquired resistance of *C*. *glabrata* to extended-spectrum triazoles, particularly fluconazole, as a consequence of widespread use of these relatively safer antifungal drugs and the haploid nature of this organism [9–12]. Although echinocandins are now preferred as first-line agents for treatment of invasive *Candida* infections [13], resistance to echinocandins in *Candida* spp. has also appeared in recent years and breakthrough invasive *C*. *glabrata* infections have been reported in patients on micafungin therapy [6, 14–19]. Resistance to polyenes has also been described in clinical *C*. *glabrata* isolates [20–24]. A multidrug-resistant *C*. *glabrata* phenotype (resistant to azoles and echinocandins) occurring in ICU and non-ICU settings has also been noted in recent years [25, 26]. Two other closely related species: *Candida nivariensis* and *Candida bracarensis*, with similar niches in humans, share many phenotypic characteristics with *C*. *glabrata* sensu stricto. *Candida nivariensis* and *C*. *bracarensis* are usually misidentified as *C*. *glabrata* based on phenotypic identification methods alone and are generally less susceptible to azoles and amphotericin B [9, 27–30]. Accurate species-specific identification of all clinical *C*. *glabrata* sensu lato isolates is thus warranted.

The three species belonging to *C*. *glabrata* complex can be accurately identified only by molecular methods including matrix assisted laser desorption ionization-time of flight mass spectrometry (MALDI-TOF MS) [9, 30, 31]. The occurrence of *C*. *nivariensis* and *C*. *bracarensis* among phenotypically identified clinical *C*. *glabrata* sensu lato isolates from different geographical settings remains unknown. This study developed a simple, low-cost multiplex PCR (mPCR) assay for rapid detection and differentiation of *C*. *nivariensis* and *C*. *bracarensis* from *C*. *glabrata* sensu stricto isolates. Furthermore, three species-specific singleplex PCR assays were also developed and the protocols were tested on a large collection of *Candida glabrata* sensu lato isolates obtained from various clinical specimens in Kuwait. The internal transcribed spacer (ITS) region of rDNA was also sequenced and rDNA sequence variations were used for determining genetic relatedness among selected isolates.

## Materials and methods

### Reference strains and clinical isolates

Reference strains or well characterized clinical isolates of *C*. *glabrata* sensu stricto (ATCC 90030 and CBS 138), *C*. *nivariensis* (CBS 9983), *C*. *bracarensis* (CBS 10154), *C*. *albicans* (ATCC 76615), *C*. *parapsilosis* (ATCC 22019), *C*. *tropicalis* (ATCC 750) and *C*. *dubliniensis* (CBS 7987), *C*. *lusitaniae* (CBS 4413), *C*. *guilliermondii* (CBS 6021), *C*. *kefyr* (ATCC 28838), *C*. *famata* (CBS 796), *Saccharomyces cerevisiae* (Kw1336/19) and *Trichosporon asahii* (Kw1728/19) were used as reference *Candida* or other yeast species. The clinical isolates used in this study were obtained from different specimens, including blood samples, from patients after obtaining verbal consent at various hospitals across Kuwait. The isolation and identification of fungal pathogens was performed as part of routine patient care and diagnostic work-up. The results obtained from the clinical isolates used in this study are presented on deidentified samples. The clinical isolates were initially identified at the hospital microbiology laboratory as *C*. *glabrata* sensu lato by the Vitek2 yeast identification system (bioMerieux, Marcy-lEtoile, France). The cultured isolates were sent to Mycology Reference Laboratory (MRL), Department of Microbiology, Faculty of Medicine, Kuwait University for further identification and antifungal susceptibility testing. A total of 440 *C*. *glabrata* sensu lato isolates, collected during 2006 to 2015, were randomly selected from the stock cultures maintained at MRL for this study. The clinical specimens yielding 440 *C*. *glabrata* sensu lato isolates used in this study are listed in S1 Table.

### Phenotypic identification

All 440 *C*. *glabrata* sensu lato isolates, initially identified by Vitek2 ID-YST system (bioMerieux, Marcy-lEtoile, France) were grown on CHROMagar Candida (Becton Dickinson, Bootle, UK) for phenotypic identification and the results were interpreted according to manufacturer’s instructions. The typical pink color of *C*. *glabrata* sensu stricto and white color of *C*. *nivariensis* and *C*. *bracarensis* [30] was used for species-specific identification of these isolates.

### Template DNA preparation and molecular identification

The genomic DNA from reference strains and clinical *C*. *glabrata* sensu lato isolates was extracted by using Gentra Puregene Yeast DNA extraction kit (Qiagen, Hilden Germany) according to kit instructions or by the rapid boiling method using Chelex-100 as described previously [32].

A simple, low-cost mPCR assay was developed for rapid molecular identification of *C*. *glabrata* sensu stricto, *C*. *nivariensis* and *C*. *bracarensis* isolates. For this purpose, three different forward primers targeting specific sequences within ITS-1 region of rDNA of the three species and one common reverse primer targeting 5.8S rRNA gene were synthesized (S2 Table). The primer sequences were selected based on multiple sequence alignment of ITS region sequences of multiple strains of all commonly encountered *Candida* species that are available from the GenBank. The mPCR should yield amplicons of ~360 bp, ~250 bp and ~180 bp (due to slight variations in the length of ITS region of rDNA) from *C*. *glabrata* sensu stricto, *C*. *nivariensis* and *C*. *bracarensis* respectively, while no amplicon is expected from other *Candida* or other yeast species. The species specificity of the combination of forward primers mCGLF, mCNIF and mCBRF together with a common reverse primer (mCGCR) for *C*. *glabrata* sensu stricto, *C*. *nivariensis* and *C*. *bracarensis*, respectively, was also indicated by BLAST searches (http://blast.ncbi.nlm.nih.gov/Blast.cgi?). The mPCR amplification was performed in a final volume of 50 μl containing 1x AmpliTaq DNA polymerase buffer I and 1 unit of AmpliTaq DNA polymerase (Applied Biosystems, Brachburg, NJ, USA), 10 pmol of mCGLF, mCNIF, mCBRF and mCGCR primers, 2 μl of template DNA and 100 μM of each dNTP. Cycling conditions included an initial denaturation at 95°C for 5 min followed by 30 cycles of 95°C for 1 min, 52°C for 30 s and 72°C for 1 min and a final extension at 72°C for 10 min. PCR products (20 μl) were run on 2% (w/v) agarose gels, as described previously [33].

### Primer design for three species-specific singleplex PCR assays

The results of mPCR were confirmed for all isolates by developing three species-specific singlelex PCR assays. For this purpose, three pairs of forward and reverse primers, each pair specific for *C*. *glabrata* sensu stricto, *C*. *nivariensis* or *C*. *bracarensis* were designed (S2 Table). Again, primer sequences were selected based on multiple sequence alignment of ITS region sequences of multiple strains of common *Candida* species available from the GenBank. Primers CGLF + CGLR, CNIF + CNIR and CBRF + CBRR should yield amplicons of ~360 bp, ~288 bp and ~299 bp (due to slight variations in the length of ITS region of rDNA) from *C*. *glabrata* sensu stricto, *C*. *nivariensis* and *C*. *bracarensis*, respectively, while no amplicon is expected from other *Candida* or other yeast species. The species specificity of CGLF + CGLR, CNIF + CNIR and CBRF + CBRR primer pairs for *C*. *glabrata* sensu stricto, *C*. *nivariensis* and *C*. *bracarensis*, respectively, was also indicated by BLAST searches (http://blast.ncbi.nlm.nih.gov/Blast.cgi?). PCR amplification was performed and the amplicons were detected as described above except that CGLF + CGLR or CNIF + CNIR or CBRF + CBRR primer pairs were used.

### PCR-sequencing of ITS region of rDNA and identification of major ITS haplotypes

The ITS region of rDNA was amplified by using panfungal ITS1 and ITS4 primers and the amplicons were sequenced by using internal sequencing (ITS1FS, ITS2, ITS3 or ITS4RS) primers, as described previously [34, 35]. The ITS region sequence for each isolate was assembled and used for BLAST searches (http://blast.ncbi.nlm.nih.gov/Blast.cgi?). Sequence identity >99% with the corresponding sequence from reference strains of *Candida* species was used for species identification. The genotypic relationship among the isolates was studied by comparing ITS region sequences. Pairwise comparisons and multiple sequence alignments were performed with CLUSTAL W2. Phylogenetic tree was constructed with MEGA software version X using the neighbor-joining method with pair-wise deletion of gaps option and the maximum composite likelihood model and the robustness of tree branches was assessed by bootstrap analysis with 1,000 replicates, as described previously [36].

## Results

### Phenotypic identification of *C*. *glabrata* complex isolates

The reference strains of *C*. *glabrata* sensu stricto (ATCC 90030), *C*. *nivariensis* (CBS 9983) and *C*. *bracarensis* (CBS 10154) yielded purple, white and white colored colonies on CHROMagar Candida, respectively, as expected. However, when 440 clinical isolates were streaked on CHROMagar candida medium, only 42 isolates produced typical purple color colonies, while 321 isolates produced light purple colonies with white tinge and the remaining 77 isolates formed completely white colonies with no noticeable purple color.

### Establishment of a multiplex PCR (mPCR) assay and analysis of clinical *C*. *glabrata* sensu lato isolates

**Fig 1:**
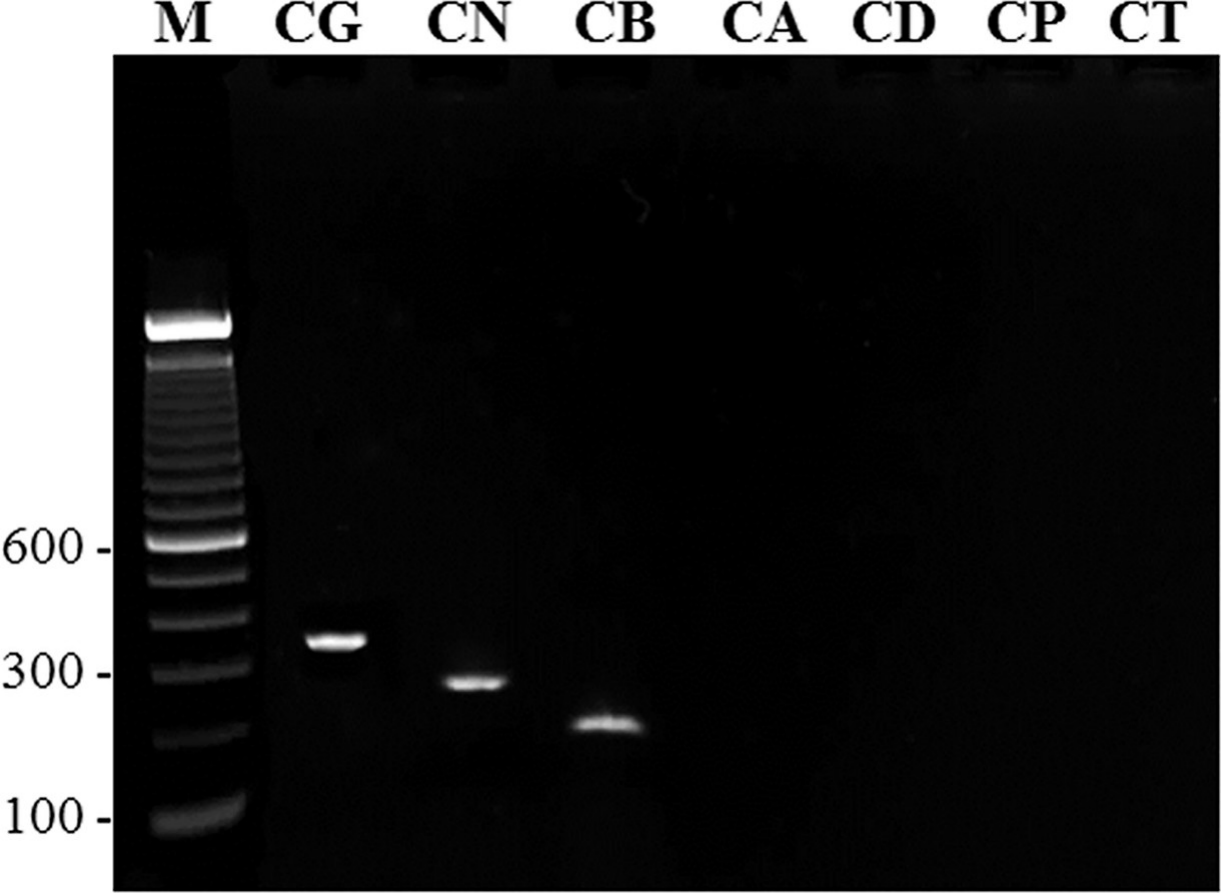
An agarose gel of amplified products obtained in mPCR using *C*. *glabrata* sensu stricto-specific (mCGLF), *C*. *nivariensis*-specific (mCNIF) and *C*. *bracarensis*-specific (mCBRF) forward and *C*. *glabrata* complex-specific (mCGCR) reverse primer with genomic DNA from reference strain of *C*. *glabrata* sensu stricto ATCC 90030 (lane CG), *C*. *nivariensis* CBS 9983 (lane CN), *C*. *bracarensis* CBS 10154 (lane CB), *C*. *albicans* ATCC 76615 (lane CA), *C*. *dubliniensis* CBS 7987 (lane CD), C. *parapsilosis* ATCC 22019 (lane CP) and *C*. *tropicalis* ATCC750 (lane CT). Lane M is 100 bp DNA ladder and the position of migration of 100 bp, 300 bp and 600 bp fragments are marked.

The mPCR amplification performed with mCGLF + mCNIF + mCBRF + mCGCR primers yielded an expected size amplicon of nearly 360 bp, 250 bp and 180 bp with genomic DNA from the reference strains of *C*. *glabrata* sensu stricto (ATCC 90030), *C*. *nivariensis* (CBS 9983) and *C*. *bracarensis* (CBS 10154), respectively (Fig 1). No amplification was obtained in mPCR with DNA from reference strains of *C*. *albicans* (ATCC 76615), *C*. *dubliniensis* (CBS 7987), *C*. *parapsilosis* (ATCC 22019) or *C*. *tropicalis* (ATCC 750) (Fig 1). No amplification was also obtained in mPCR with DNA prepared from *Candida lusitaniae* (CBS 4413), *Candida guilliermondii* (CBS 6021), *Candida kefyr* (ATCC 28838), *Candida famata* (CBS 796), *Saccharomyces cerevisiae* (Kw1336/19), *Trichosporon asahii* (Kw1728/19) or from human cells, as expected. Ten-fold serial dilutions were made and the minimum detection limit for a positive test in the mPCR assay was found to be 960 pg, 500 pg and 512 pg of genomic DNA from *C*. *glabrata*, *C*. *nivariensis* and *C*. *bracarensis*, respectively. When DNA prepared from clinical *C*. *glabrata* sensu lato isolates was used, all 440 isolates yielded an amplicon of ~360 bp only which is characteristic of *C*. *glabrata* sensu stricto strains. None of the clinical isolates yielded an amplicon of 250 bp (characteristic of *C*. *nivariensis*) or 180 bp (characteristic of *C*. *bracarensis*). Thus mPCR data showed lack of detection of *C*. *nivariensis* and *C*. *bracarensis* among 440 clinical *C*. *glabrata* sensu lato isolates in Kuwait.

### Establishment of three species-specific singleplex PCR assays and analysis of clinical *C*. *glabrata* sensu lato isolates

The three singleplex PCR assays (S1 Fig, panels A-C) correctly identified the three target species and did not show any cross-reaction with other *Candida*/yeast species, as expected. When DNA isolated from 440 clinical *C*. *glabrata* sensu lato isolates was tested, an amplicon of ~360 bp was obtained in *C*. *glabrata* sensu stricto-specific singleplex PCR assay only and thus confirmed mPCR data.

### ITS haplotypes among *C*. *glabrata* isolates

**Fig 2:**
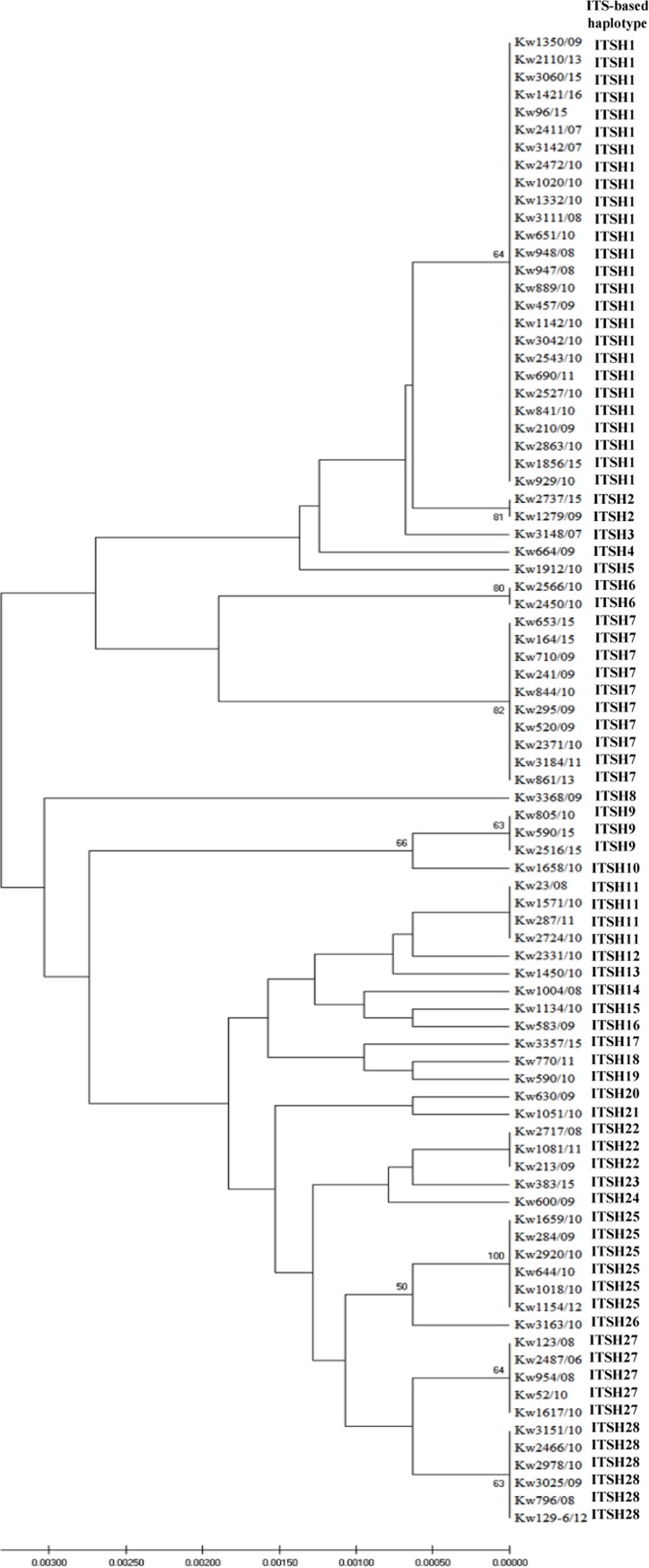
Neighbor-joining tree based on DNA sequence data for the ITS region of rDNA from 85 *C*. *glabrata* sensu stricto isolates. The bootstrap frequencies (>50%) on the node branches are shown. The ITS haplotype (ITSH) arbitrarily assigned for each isolate is also shown in the right hand column.

DNA sequencing data for the ITS region of rDNA from 85 randomly selected clinical *C*. *glabrata* isolates from Kuwait showed >99% similarity with sequences from reference *C*. *glabrata* sensu stricto strains ATCC 90030 and CBS 138 and were distinct from sequences of reference *C*. *nivariensis* and *C*. *bracarensis* strains. Multiple sequence alignment was carried out to compare the ITS region sequences. Based on ITS region sequence data, 28 distinct haplotypes (arbitrarily assigned as ITSH1 to ITSH28) were identified among 85 clinical *C*. *glabrata* isolates from Kuwait The discriminatory power for the ITS-based fingerprinting method was 0.88. Eighteen of 85 (21%) isolates belonged to unique ITS haplotypes while 67 isolates belonged to 10 cluster haplotypes with each cluster containing 2–26 isolates (Fig 2). The most common haplotype was ITSH1 shared among 26 (31%) isolates followed by ITSH7 among 10 (12%) isolates and ITSH25 and ITSH28 shared among six (7%) isolates each (Fig 2). The ITS-based fingerprinting data thus showed considerable genotypic heterogeneity among clinical *C*. *glabrata* sensu stricto isolates in Kuwait. There was no association of specific/predominant ITS haplotypes with the specimen types of *C*. *glabrata* isolates. The ITS region sequence data have been submitted to European Molecular Biology Laboratory (EMBL) database under accession numbers LS398112 to LS398120, LS398122, LS398123, LT837719 to LT837724 and LT837726 to LT837744.

## Discussion

*Candida nivariensis* and *C*. *bracarensis* generally exhibit lower susceptibility to triazoles and amphotericin B than *C*. *glabrata* sensu stricto isolates warranting species-specific identification of all clinical *C*. *glabrata* sensu lato isolates [9, 27–30, 37]. All 440 *C*. *glabrata* isolates used in this study were first tested on CHROMagar Candida. Although reference strains of *C*. *glabrata* sensu stricto (purple), *C*. *nivariensis* (creamy white) and *C*. *bracarensis* (creamy white) produced expected results as described previously [27], production of creamy white colonies on CHROMagar Candida is not restricted for *C*. *nivariensis* and *C*. *bracarensis* strains only as *Candida norvegensis*, *C*. *inconspicua* and some strains of *C*. *glabrata* also produce creamy white colonies on this medium [30, 38]. Similar to the results reported by Bishop et al. [38], only 363 of 440 (82.5%) isolates produced completely or partially purple colonies while 77 (17.5%) isolates produced creamy white colonies. The occurrence of creamy white colonies was not restricted to any specific specimen type. Lockhart et al [30] also reported that 11 of 14 isolates producing creamy white colonies in their study were subsequently identified as *C*. *glabrata* sensu stricto strains by two molecular tests. Thus creamy white colonies on CHROMagar Candida is not diagnostic for the identification of *C*. *nivariensis* and *C*. *bracarensis* strains.

In this study, we have also developed a simple, low-cost mPCR assay for accurate identification of *C*. *glabrata* sensu stricto, *C*. *nivariensis* and *C*. *bracarensis* isolates in a single PCR assay. The accuracy of mPCR assay was tested by using reference strains of several *Candida*/other yeast species. All 440 clinical *C*. *glabrata* sensu lato isolates analyzed in this study were identified as *C*. *glabrata* sensu stricto by mPCR. The mPCR-based identification of all isolates was also confirmed by a *C*. *glabrata* sensu stricto-specific singleplex PCR assay. The mPCR assay developed here can be completed within 4 hours using basic PCR and gel electrophoresis equipment that are readily available in clinical microbiology laboratories and will only cost ~2 US$ per sample (using Chelex-100-based boiling method for DNA extraction and excluding the cost of culture and personnel time).

Several molecular methods have been described previously for species-specific identification of *C*. *glabrata* sensu stricto, *C*. *nivariensis* and/or *C*. *bracarensis* isolates [39–44]. Of these, the mPCR assays described by Romeo et al. [41] and Arastehfar et al. [43] are very similar to our assay except that longer amplicons were obtained in their study. The PCR protocols yielding smaller amplicons are generally preferred [45, 46]. The larger amplicon size (~450 bp) for *C*. *glabrata* in SeptiFast real-time PCR assays yielded false-nagative results from candidemia patients in some studies even when the blood cultures yielded *C*. *glabrata* [47–49]. Singleplex PCR assays have also been described for the detection of *C*. *glabrata* sensu stricto and *C*. *nivariensis* [39, 40], however, they (including those developed in this study) are time consuming as they require three separate PCR reactions for each isolate. Although the singleplex PCR assay described by Enache-Angoulvant et al., [42] can potentially detect all the three species in a single test, the primers are not specific as amplicons were also obtained from *S*. *cerevisiae* and few other close relatives [42]. Additionally, poor resolution in agarose gels due to small (~100 bp) differences among larger (~1000 bp) amplicons and variations in the length of intronic sequences used as target among the isolates may lead to misidentification in some cases. The PNA-FISH is labor intensive and time consuming and species-specific probes have to be tested for all phenotypically identified *C*. *glabrata* sensu lato isolates [44]. Although rapid identification of *C*. *nivariensis* and *C*. *bracarensis* is possible with MALDI-TOF MS analysis, the requirement for fresh cultures often necessitates sub-culturing for obtaining interpretable results and these two species are included in the database of only the Bruker Daltonics Biotyper system but not in the database of the Biomerieux Vitek MS system [31, 50, 51].

The ITS-based fingerprinting data showed considerable genotypic heterogeneity as 28 distinct haplotypes were identified among 85 isolates. Eighteen isolates belonged to unique haplotypes while 67 isolates clustered in 10 haplotypes. The discriminatory power for the ITS-based fingerprinting method was 0.88 which is nearly same as the discriminatory power of the six-loci-based multilocus sequence typing (MLST) scheme for *C*. *glabrata* [52]. Fingerprinting of a global collection of 109 *C*. *glabrata* isolates by MLST yielded only 30 sequence types (STs) [52]. Fingerprinting studies for other *Candida* species have yielded variable results. While the ITS region sequences of 24 *C*. *parapsilosis* sensu stricto isolates analyzed so far from Kuwait were identical, three haplotypes were identified among 19 isolates of *C*. *orthopsilosis*, a species very closely related to *C*. *parapsilosis* sensu stricto [32, 53]. Likewise, the ITS region sequences among *C*. *albicans* are highly similar as only four haplotypes have been identified among a large collection of isolates so far, nine haplotypes have been detected in clinical isolates of the sister species, *C*. *dubliniensis* [36, 54–56]. The highest variability among ITS region sequences has been noted recently among *C*. *lusitaniae* strains as nine haplotypes were detected among 13 clinical isolates [57]. Taken together, the ITS region of rDNA presents as an attractive target and has the potential for improving the discriminatory power of the MLST scheme [52] for routine fingerprinting of clinical *C*. *glabrata* sensu stricto isolates.

Although we analyzed a large (n = 440) and diverse collection of clinical *C*. *glabrata* sensu lato isolates, *C*. *nivariensis* and *C*. *bracarensis* were not detected implying that these two cryptic species are not clinically important in Kuwait. The findings are consistent with a recent study that reported a low prevalence of 0.12% for *C*. *nivariensis* and 0.01% for *C*. *bracarensis* among phenotypically identified *C*. *glabrata* strains [9]. A search of published studies was carried out to determine which clinical specimens or ethnic background of patients may be more suitable for the isolation of these rare yeast pathogens and the results are summarized in Table 1. In total, 11 case reports and 18 research studies were identified which either reported the isolation or lack of isolation of *C*. *nivariensis* and/or *C*. *bracarensis*. *C*. *nivariensis* has a wider global distribution as it has been isolated from several countries in Europe, Asia (including Iran in the Middle East), Australia and South America while *C*. *bracarensis* has mainly been isolated from Canada, USA and Mexico in North America, some European countries, China and Argentina [27–30, 37, 44, 51, 58–79]. Surprisingly, both species were isolated simultaneously from only two (China and Argentina) countries [37, 51, 76]. Altogether, eight countries/geographical locations (including Kuwait) involving 2560 *C*. *glabrata* sensu lato isolates failed to identify the presence of either *C*. *nivariensis* or *C*. *bracarensis* indicating that these species are rare yeast pathogens in some countries/geographical locations [30, 68, 69, 71, 77].

**Table 1:** Summary of published studies/case reports and screening methods used for the identification of *C*. *nivariensis* and *C*. *bracarensis* among *C*. *glabrata* sensu lato isolates.

No.	Type of study	Year of publication	Country/Region	No. of isolates screened	No. (%) of isolates identified as			Identification method(s)		Reference
					*CG*	*CN*	*CB*	Preliminary test	Confirmatory test	
1	Case reports	2005	Spain	NA	NA	3	-	CHROMagar	DNA sequencing	Alcoba-Flo´rez et al., 2005 [27]
2	Case report	2006	Portugal/UK	NA	NA	-	2	-	DNA sequencing	Correia et al., 2006 [28]
3	Case report	2007	Japan	NA	NA	1	-	-	DNA sequencing	Fujita et al., 2007 [58]
4	Case report	2008	Indonesia	NA	NA	1	-	CHROMagar/Auxacolor2	DNA sequencing	Wahyuningsih et al., 2008 [59]
5	Case reports	2008	UK	NA	NA	16	-	CHROMagar/Auxacolor2/ API 20C	DNA sequencing	Borman et al., 2008 [29]
6	Case report	2010	Canada	NA	NA	-	2	CHROMagar	DNA sequencing	Warren et al., 2010 [60]
7	Case report	2013	Spain	NA	NA	1	-	CHROMagar	DNA sequencing	Lopez-Soria et al., 2013 [61]
8	Case reports	2013	UK	NA	NA	5	-	CHROMagar	DNA sequencing/MALDI-TOF	Gorton et al., 2013 [62]
9	Case reports	2016	Spain	NA	NA	4	-	CHROMagar	DNA sequencing/MALDI-TOF	Aznar-Marin et al., 2016 [63]
10	Case report	2016	Brazil/Rio de Janeiro	NA	NA	1	-	CHROMagar/Vitek2		Figueiredo-Carvalho et al., 2016 [64]
11	Case report	2018	Mexico	NA	NA	-	1	API 20C	DNA sequencing	Treviño-Rangel et al., 2018 [65]
12	Research/screening	2008	USA/Baltimore	137	134 (98)	0	3 (2)	CHROMagar/Vitek2	PNA FISH/DNA sequencing	Bishop et al., 2008 [44]
13	Research/screening	2009	North America	838	836 (99.8)	0	2 (0.2)	CHROMagar	PCR/PNA FISH	Lockhart et al., 2009 [30]
			South America	133	133 (100)	0	0	CHROMagar	PCR/PNA FISH	
			Europe	400	400 (100)	0	0	CHROMagar	PCR/PNA FISH	
			Asia	111	111 (100)	0	0	CHROMagar	PCR/PNA FISH	
			Australia	42	41 (97.6)	1 (2.4)	0	CHROMagar	PCR/PNA FISH	
			Africa	71	71 (100)	0	0	CHROMagar	PCR/PNA FISH	
14	Research/screening	2010	India	363	361 (99.5)	2 (0.5)	0	CHROMagar	DNA sequencing	Chowdhary et al., 2010 [66]
15	Research/screening	2011	Spain	143	140 (98)	0	3 (2)	NS	DNA sequencing	Cuenca-Estrella et al., 2011 [67]
16	Research/screening	2011	Denmark	133	133 (100)	0	0	NS	PCR-RFLP/PNA FISH	Mirhendi et al., 2011 [68]
17	Research/screening	2012	Spain	158	158 (100)	0	0	NS	PCR/DNA sequencing	Pemán et al, 2012 [69]
18	Research/screening	2013	India	100	96 (96)	4 (4)	0	CHROMagar/Vitek2	PCR/DNA sequencing	Sharma et al., 2013 [70]
19	Research/screening	2013	Italy	1000	1000 (100)	0	0	CHROMagar/ID32C	mPCR	Esposto et al., 2013 [71]
20	Research/screening	2014	China/Shenzhen	301	293 (97.3)	7 (2.3)	1 (0.3)	CHROMagar/API 20C	DNA sequencing	Li et al., 2014 [37]
21	Research/screening	2014	Poland	224	211 (94)	13 (6)	0	CHROMagar/ID32C	DNA sequencing	Swoboda-Kopec et al., 2014 [72]
22	Research/screening	2014	Malaysia	185	183 (99)	2 (1)	0	CHROMagar	PCR (RPL31 gene)/DNA sequencing	Tay et al., 2014 [73]
23	Research/screening	2015	China/Shanghai	NA	NA	1	-	CHROMagar/API 20C	DNA sequencing	Feng et al., 2015 [74]
24	Research/screening	2016	Argentina/Buenos Aires	117	114 (97)	3 (3)	0	CHROMagar	PCR (RPL31 gene)/DNA sequencing	Morales-López et al., 2016 [75]
25	Research/screening	2017	Argentina/Buenos Aires	122	114 (93.5)	5 (4)	3 (2.5)	NS	mPCR/DNA sequencing	Morales-López et al., 2017 [76]
26	Research/screening	2017	China/Beijing	960	947 (98.6)	12 (1.3)	1 (0.1)	Vitek2	DNA sequencing	Hou et al., 2017 [51]
27	Research/screening	2018	Spain	114	114 (100)	0	0	CHROMagar/ID32C	mPCR	Miranda-Cadena et al., 2018 [77]
28	Research/screening	2018	Poland	353	352 (99.7)	0	1 (0.3)	CHROMagar/Vitek2	mPCR/DNA sequencing	Małek et al., 2018 [78]
29	Research/screening	2019	Iran	213	209 (98)	4 (2)	0	CHROMagar/mPCR	DNA sequencing/MALDI-TOF	Arastehfar et al., 2019 [79]
30	Research/screening	2019	Kuwait	440	440 (100)	0	0	CHROMagar/Vitek2	PCR/mPCR/DNA sequencing	This study

CG, *C*. *glabrata* sensu stricto; CN, *C*. *nivariensis*; CB, *C*. *bracarensis*; mPCR, multiplex PCR; PNA-FISH, peptide nucleic acid-fluorescent in situ hybridization; NS, not specified; NA, not applicable

The clinical specimens yielding *C*. *nivariensis* or *C*. *bracarensis* among ~6500 *C*. *glabrata* sensu lato isolates screened so far in various studies are listed in Table 2. The data show that bloodstream, vaginal specimens and respiratory specimens are more likely to yield *C*. *nivariensis* or *C*. *bracarensis*.

**Table 2:** Number of *C*. *nivariensis* and/or *C*. *bracarensis* isolates identified in various clinical specimens among ~6500 *C*. *glabrata* sensu lato isolates screened so far.

Clinical specimens	No. of *C*. *nivariensis*	No. of *C*. *bracarensis*
screened	isolates detected	isolates detected
Blood	22	5
Vaginal swab/exudate	12	3
Respiratory tract/oral cavity/sputum	14	2
Ascitic fluid	3	0
Urine/renal sample	2	0
Stool	0	2
Abscess/pus	2	2
Pleural fluid	1	1
Cerebrospinal fluid	1	0
Catheter exudate/exit site swab	1	1
Surgical site swab	1	0
CAPB dialysis fluid	1	0
Unspecified	6	0
**Total**	**66**	**16**

CAPB, continuous ambulatory peritoneal bag

In conclusion, we have described a simple, cost effective mPCR assay by using highly conserved rDNA sequences for rapid detection and differentiation of *C*. *nivariensis* and *C*. *bracarensis* from *C*. *glabrata* sensu stricto isolates. The method involves single-tube PCR amplification followed by agarose gel electrophoresis for detection of species-specific amplicons. Only basic PCR and gel electrophoresis equipment are needed which are readily available in most mycology laboratories and the requirement for a minimal amount of genomic DNA allows the whole procedure to be completed within 4 hours. The mPCR accurately identified all 440 *C*. *glabrata* sensu stricto isolates. Furthermore, our study highlights lack of detection of *C*. *nivariensis* and *C*. *bracarensis* from Kuwait further supporting that *C*. *nivariensis* and *C*. *bracarensis* are rare yeast pathogens in some geographical locations. Considerable genotypic diversity among *C*. *glabrata* sensu stricto isolates was also indicated by rDNA sequence analyses.

Our study has few limitations. Only one reference strain was available for *C*. *nivariensis* and *C*. *bracarensis* during specificity testing studies and an internal PCR control was not included. Also, misidentification of one of the *Candida* species may occur in the event of mixed colonies as it will not be amplified by mPCR.

## Supporting information

S1 Table Clinical source of 440 *C*. *glabrata* sensu lato isolates used in this study. (DOCX)

S2 Table Nucleotide sequences and specific purpose of primers used in PCR-amplification of various genomic regions of *C*. *glabrata* sensu lato isolates and the expected sizes of amplicons in base pairs (bp). (DOCX)

S1 Fig Agarose gel of amplified products obtained in PCR using *C*. *glabrata*-specific (panel A), *C*. *nivariensis*-specific (panel B) and *C*. *bracarensis*-specific (panel C) primers with genomic DNA from reference strain of *C*. *glabrata* (lane CG), *C*. *nivariensis* (lane CN), *C*. *bracarensis* (lane CB), *C*. *albicans* (lane CA), *C*. *dubliniensis* (lane CD), *C*. *parapsilosis* (lane CP) and *C*. *tropicalis* (lane CT). Lane M is 100 bp DNA ladder.
(DOCX)
