# Supplementary material for: Lack of detection of Candida nivariensis and Candida bracarensis among 440 clinical Candida glabrata sensu lato isolates in Kuwait
Source: PLoS One. 2019 Oct 16;14(10):e0223920. doi: 10.1371/journal.pone.0223920 (PMC6795469; doi:10.1371/journal.pone.0223920)
Supplement: S1 Table — (DOCX) [file pone.0223920.s001.docx]

**S1 Table**. **Clinical source of 440 *C. glabrata* sensu lato isolates used in this study**

| **Clinical source** | **No. of isolates** |
| --- | --- |
| Urine | 138 |
| Sputum | 84 |
| Endotracheal aspirate | 59 |
| Blood | 33 |
| Oral/throat swab | 29 |
| Vaginal swab | 12 |
| Wound/pus swab | 12 |
| Broncho alveolar lavage | 9 |
| Pleural fluid | 7 |
| Gastric tissue/fluid | 7 |
| Skin swab | 6 |
| Cavitary fluid | 6 |
| Bedsore swab | 5 |
| Rectal swab | 4 |
| ^*^Others | 29 |
| Total | 440 |

*Other specimens included; tissue sample; ileostomy; axilla and groin swab; central venous catheter tip and abdominal drain fluid
