# Supplementary material for: Lack of detection of Candida nivariensis and Candida bracarensis among 440 clinical Candida glabrata sensu lato isolates in Kuwait
Source: PLoS One. 2019 Oct 16;14(10):e0223920. doi: 10.1371/journal.pone.0223920 (PMC6795469; doi:10.1371/journal.pone.0223920)
Supplement: S2 Table — (DOCX) [file pone.0223920.s002.docx]

**S2 Table. Nucleotide sequences and specific purpose of primers used in PCR-amplification of various genomic regions of *C. glabrata* sensu lato isolates and the expected sizes of amplicons in base pairs (bp)**

| Primer | Nucleotide sequence | Purpose | Amplicon | Reference |
| --- | --- | --- | --- | --- |
|  |  |  | size* |  |
| mCGLF | 5'-CGGTTGGTGGGTGTTCTGC-3' | Forward multiplex primer for *C. glabrata* sensu stricto | ~360 bp | Romeo et al., 2009 [40] |
| mCNIF | 5'-GAGGAGTTTGTATCTTTCAACTT-3' | Forward multiplex primer for *C. nivariensis* | ~250 bp | This study |
| mCBRF | 5'-GGGACGGTAAGTCTCCCG-3' | Forward multiplex primer for *C. bracarensis* | ~180 bp | Romeo et al., 2009 [40] |
| mCGCR | 5'-CACGGAATTCTGCAATTCACA-3' | Reverse multiplex primer for *C. glabrata* sensu lato |  | This study |
| CGLF | 5'-ACTTTACTACTATTCTTTTGTTCG-3' | Forward species-specific primer for *C. glabrata* sensu stricto | ~360 bp | This study |
| CGLR | 5'-CAATTTCAAGTTAACTCAAAAAC-3' | Reverse species-specific primer for *C. glabrata* sensu stricto |  | This study |
| CNIF | 5'-TCTACTTTGTCAAAACCAAATTC-3' | Forward species-specific primer for *C. nivariensis* | ~288 bp | This study |
| CNIR | 5'-ACCATGCACGTCCGCATAAA-3' | Reverse species-specific primer for *C. nivariensis* |  | This study |
| CBRF | 5'-TATTTACAAACTTTGTCAGAACTTA-3' | Forward species-specific primer for *C. bracarensis* | ~299 bp | This study |
| CBRR | 5'-AGACTCCAAATGTCCCCACTA-3' | Reverse species-specific primer for *C. bracarensis* |  | This study |

*Amplicon sizes (in base pairs, bp) are based on various combinations of forward primers with their reverse primer. The amplicon sizes could vary slightly due to variations in the length of ITS-1 and/or ITS-2 among various strains of different *Candida* species.
