# Supplementary material for: Lack of detection of Candida nivariensis and Candida bracarensis among 440 clinical Candida glabrata sensu lato isolates in Kuwait
Source: PLoS One. 2019 Oct 16;14(10):e0223920. doi: 10.1371/journal.pone.0223920 (PMC6795469; doi:10.1371/journal.pone.0223920)

**S1 Fig.** **Agarose gel of amplified products obtained in PCR using *C. glabrata* sensu stricto-specific (panel A), *C. nivariensis*-specific (panel B) and *C. bracarensis*-specific (panel C) primers with genomic DNA from reference strain of *C. glabrata* sensu stricto (lane CG), *C. nivariensis* (lane CN), *C. bracarensis* (lane CB), *C. albicans* (lane CA), *C. dubliniensis (*lane CD), C. *parapsilosis* (lane CP) and *C. tropicalis* (lane CT).** Lane M is 100 bp DNA ladder and the position of migration of 100 bp, 300 bp and 600 bp fragments are marked.


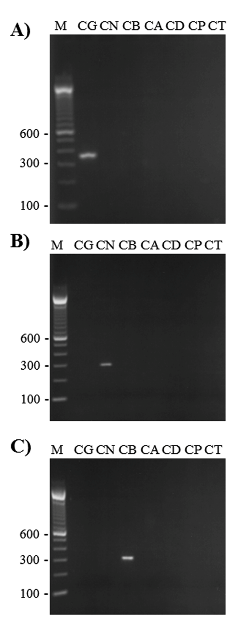

Supplement: S1 Fig — Agarose gel of amplified products obtained in PCR using C. glabrata-specific (panel A), C. nivariensis-specific (panel B) and C. bracarensis-specific (panel C) primers with genomic DNA from reference strain of C. glabrata (lane CG), C. nivariensis (lane CN), C. bracarensis (lane CB), C. albicans (lane CA), C. dubliniensis (lane CD), C. parapsilosis (lane CP) and C. tropicalis (lane CT). Lane M is 100 bp DNA ladder. (DOCX) [file pone.0223920.s003.docx]
